# ATRX is required for maintenance of the neuroprogenitor cell pool in the embryonic mouse brain

**DOI:** 10.1242/bio.20148730

**Published:** 2014-11-13

**Authors:** Kieran Ritchie, L. Ashley Watson, Benjamin Davidson, Yan Jiang, Nathalie G. Bérubé

**Affiliations:** Departments of Paediatrics and Biochemistry, Children's Health Research Institute, University of Western Ontario, Victoria Research Laboratories, 800 Commissioners Road East, London, ON N6C 2V5, Canada

**Keywords:** ATRX, Cell division, Chromatin, Cortical lamination, Neurodevelopment

## Abstract

Mutations in the alpha-thalassemia mental retardation X-linked (*ATRX*) gene cause a spectrum of abnormalities including intellectual disability, developmental delay, seizures, and microcephaly. The ATRX protein is highly enriched at heterochromatic repetitive sequences adjacent to the centromere, and ATRX depletion results in chromosome congression, segregation, and cohesion defects. Here, we show that Cre-mediated inactivation of *Atrx* in the embryonic mouse (*Mus musculus*) brain results in expansion of cerebral cortical layer VI, and a concurrent thinning of layers II–IV. We observed increased cell cycle exit during early-mid neurogenesis, and a depletion of apical progenitors by late neurogenesis in the *Atrx*-null neocortex, explaining the disproportionate layering. Premature differentiation was associated with an increased generation of outer radial glia (oRG) and TBR2-expressing basal progenitors, as well as increased generation of early-born post-mitotic projection neurons. *Atrx* deletion also reduced the fidelity of mitotic spindle orientation in apical progenitors, where mutant cells were often oriented at non-parallel angles of division relative to the ventricular surface. We conclude that ATRX is required for correct lamination of the mouse neocortex by regulating the timing of neuroprogenitor cell differentiation.

## INTRODUCTION

The ATRX protein belongs to a family of SWI/SNF2-type chromatin remodeling proteins. Hypomorphic mutations in the *ATRX* gene are associated with both syndromic and non-syndromic forms of X-linked intellectual disability, including the ATR-X syndrome (Costa et al., 2006; Gibbons et al., 1995). ATRX utilizes the energy of ATP hydrolysis to disrupt histone-DNA interactions, exhibits DNA translocase activity, and partners with DAXX to deposit the histone variant H3.3 at pericentromeric and telomeric heterochromatin (Drané et al., 2010; Goldberg et al., 2010; Lewis et al., 2010; Mitson et al., 2011; Xue et al., 2003). ATRX becomes hyperphosphorylated at the onset of the mitotic phase of the cell cycle (Bérubé et al., 2000), and has been shown to be required for normal chromosome alignment and cohesion (Baumann et al., 2010; De La Fuente et al., 2004; Ritchie et al., 2008).

Inactivation of *Atrx* in the developing mouse forebrain leads to reduced forebrain size, hippocampal dysgenesis, genomic instability, and elevated p53-dependent neuronal apoptosis (Bérubé et al., 2005; Seah et al., 2008; Watson et al., 2013). Inactivation of p53 rescues neuroprogenitor cell (NPC) death; however, only a partial recovery of cortical size is observed at birth, indicating that additional mechanisms contribute to the *Atrx*-dependent cortical phenotype in this model (Seah et al., 2008).

The mature mammalian cerebral cortex is laminated into six stratified layers, each populated by distinct neuronal subtypes (Campbell, 2005). Newly born neurons migrate radially away from the lateral ventricles along a scaffold of radial glia projections to their final cortical destinations in an inside-out fashion, with the earliest born neurons populating the deep cortical layers (V–VI), and late-born neurons being found in the superficial layers (II–IV) (Rakic, 1974).

The mechanism underlying cortical neurogenesis demands further clarification, but the most current model implicates the mitotic spindle angle as a determinant of cell fate. Inheritance of the basal process that contacts the pial surface results in a progenitor cell fate, whereas the daughter cell that does not inherit the basal process becomes a neuron-committed cell that delaminates and migrates basally as described above (Konno et al., 2008). Recent work has uncovered an additional type of progenitor, the outer radial glial (oRG) cell, which is produced from very oblique division angles in which the daughter cell inheriting the basal process inherits no portion of the apical membrane (Shitamukai et al., 2011; Wang et al., 2011). These oRG progenitors migrate just past the subventricular zone (SVZ), where they divide asymmetrically. Models that randomize the mitotic spindle angle have produced contradictory results. For instance, some models have shown spindle randomization to increase the oRG population (Shitamukai et al., 2011), others have shown increased SVZ basal progenitors (Postiglione et al., 2011), and others an early increase in neurogenesis causing progenitor depletion and microcephaly (Bachmann et al., 2010).

In the present report, we show that embryonic forebrain-specific deletion of *Atrx* results in irregular postnatal neocortical layering due to increased cell cycle exit during early neurogenesis resulting in the increased production of oRG progenitors, basal progenitors, and early-born post-mitotic neurons. This initial boost in cell cycle exit was associated with randomization of apical progenitor mitotic spindle angles and resulted in a gradual depletion of the apical progenitor pool by late neurogenesis, explaining the reduced number of upper layer cortical neurons in the postnatal mutant cortex. Thus, ATRX influences the timing and balance of NPC proliferation and differentiation.

## RESULTS AND DISCUSSION

### Randomized mitotic spindle orientation in *Atrx*-null apical progenitors

**Fig. 1.**
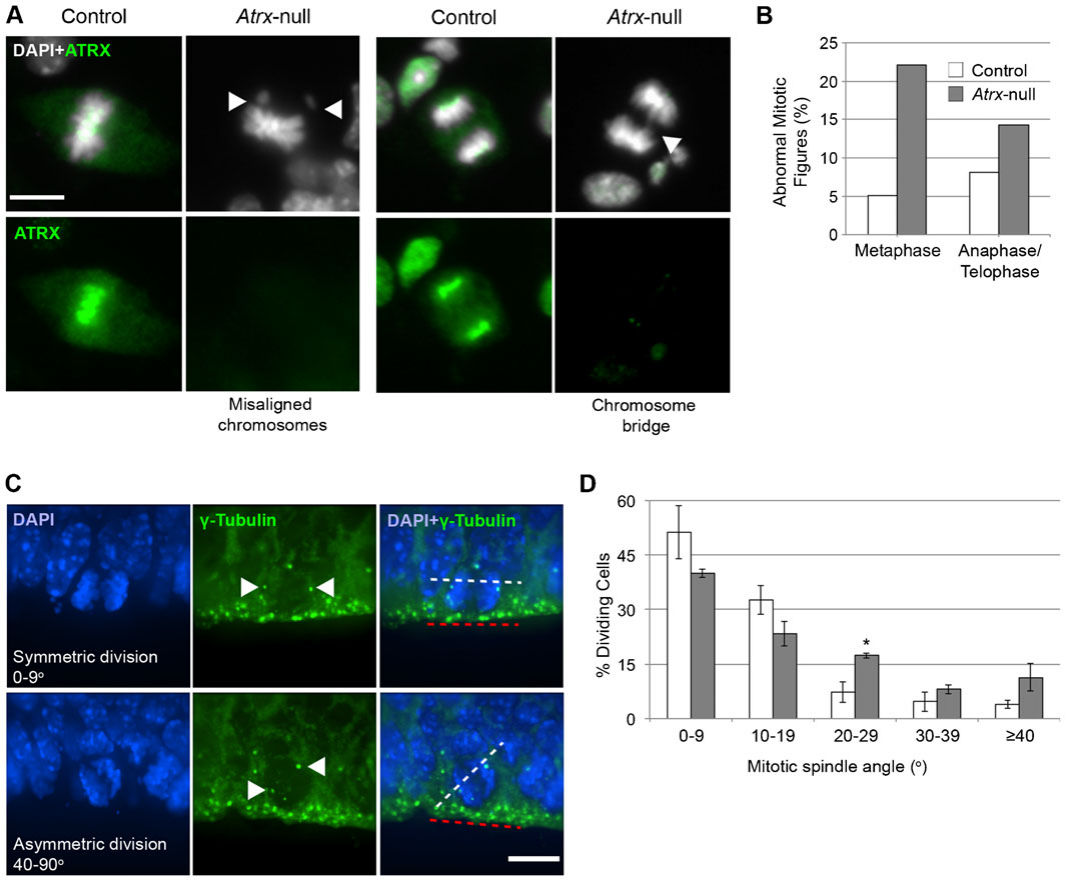
*Atrx*-null NPCs exhibit mitotic defects *in vitro* and altered cell division axis *in vivo*. (A) ATRX immunostaining in NPC cultures. DAPI was used as a counterstain. Arrowheads indicate abnormal mitotic figures. Scale bar: 10 µm. (B) Mitotic cells were scored for presence of misaligned chromosomes at the metaphase plate and chromosome bridges or lagging chromosomes at anaphase/telophase (control *n* = 161; *Atrx*-null *n* = 114, from 3–4 embryos). (C) γ-Tubulin immunostaining in E13.5 control and *Atrx*-null cortex. Arrowheads indicate the centrosomes. The axis of NPC division at the neuroepithelial surface was measured using the axis between centrosomes (white dashed line) relative to the neuroepithelial surface (red dashed line). Scale bar: 10 µm. (D) Angle of division was scored in control and *Atrx*-null cortex (*n* = 150 cells; 3 pairs). Data expressed as mean ± S.E.M. *P<0.05.

Given the recent emphasis on mitotic spindle orientation in cortical neurogenesis and our previous finding of mitotic abnormalities in ATRX-depleted human cells (Ritchie et al., 2008), we investigated whether mouse *Atrx*-null NPCs exhibit similar mitotic defects. Control and *Atrx*-null NPC cultures were stained with DAPI two days after plating, at which time the NPCs are actively dividing (Fig. 1A). Mitotic figures were identified by their characteristic condensed chromatin morphologies, and scored for evidence of misaligned chromosomes during metaphase as well as chromatin bridges during anaphase/telophase. Embryonic NPCs isolated from *Atrx*-null cortices exhibited a high level of mitotic abnormalities compared to control cells (Fig. 1A,B), indicating that ATRX is required for normal chromosome dynamics during mitotic cell division of cortical NPCs.

We next measured the angle of the mitotic spindle axis relative to the ventricular surface in E13.5 control and *Atrx*-null apical progenitor cells. For this analysis, the centrosomal marker γ-tubulin was used as a marker of the spindle poles (Fig. 1C). In apical progenitors, 84% of mitotic spindles were oriented in a near-parallel (0–20°) angle to the apical surface while only 63% of *Atrx*-null mitotic cells were scored in this category (Fig. 1D). Rather, mitotic apical progenitors in the *Atrx*-null forebrain were more frequently oriented in non-parallel angles (>20°) relative to the apical surface (Fig. 1D). This result suggests that ATRX loss causes abnormal mitosis and randomization of the mitotic spindle angle in apical progenitor cells, which may explain the increase in differentiative divisions observed in the *Atrx*-null cortex during early neurogenesis.

It has recently been shown that ATRX partners with DAXX to deposit the histone variant H3.3 at telomeric repeats and pericentromeric heterochromatin (Drané et al., 2010; Goldberg et al., 2010). Reduced ATRX-dependent H3.3 deposition at pericentromeric chromatin inhibited the expression of pericentromeric transcripts (Drané et al., 2010). Failure to correctly incorporate histone H3.3 may thus disrupt the integrity or function of pericentromeric heterochromatin to promote correct alignment and separation of sister chromatids during mitosis, perhaps explaining the mitotic defects observed in *Atrx*-null NPCs.

### Premature neurogenesis in the *Atrx*-null cortex results in depletion of the progenitor pool by late neurogenesis

**Fig. 2.**
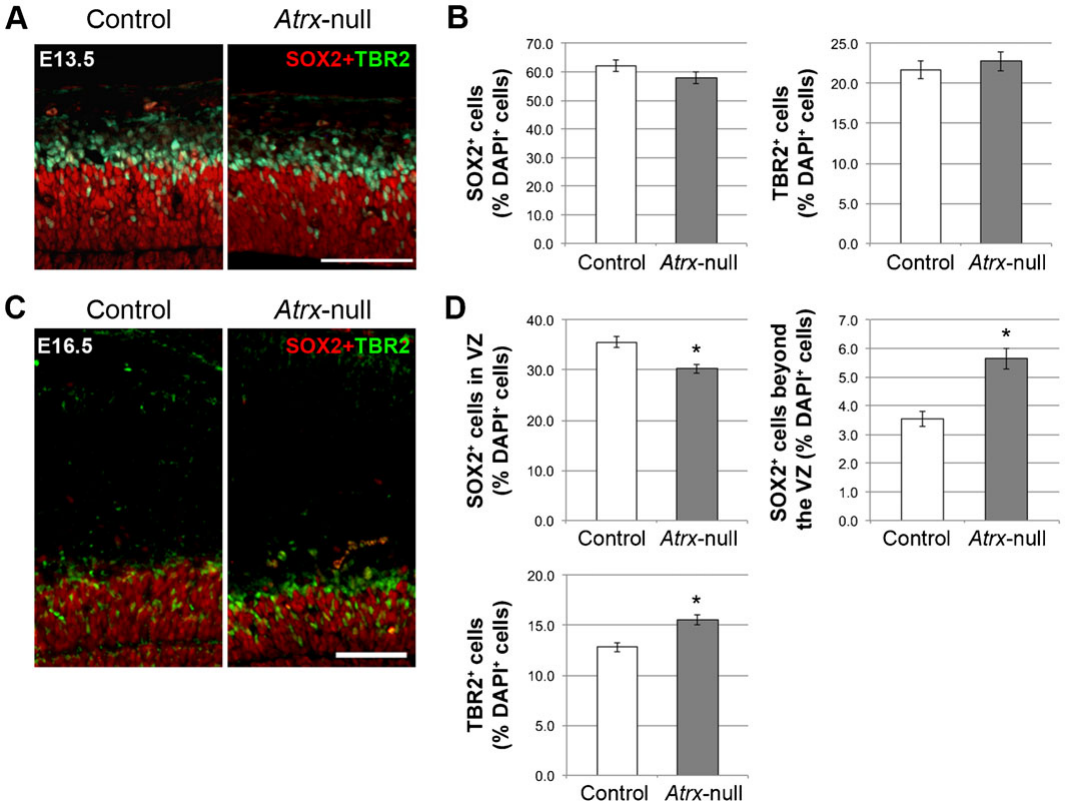
The apical progenitor pool becomes progressively depleted by late neurogenesis in the *Atrx*-deficient cortex. (A) SOX2 and TBR2 immunostaining in E13.5 control and *Atrx*-null cortex. Scale bar: 100 µm. (B) Quantification of SOX2^+^ and TBR2^+^ cells as a percentage of DAPI^+^ cells in E13.5 control and *Atrx*-null cortex (*n* = 3). (C) SOX2 and TBR2 immunostaining in E16.5 control and *Atrx*-null cortex. Scale bar: 100 µm. (D) Quantification of SOX2^+^ (both within the VZ and beyond the VZ) and TBR2^+^ cells as a percentage of total DAPI^+^ cells in E16.5 control and *Atrx*-null cortex (*n* = 3). Data expressed as mean ± S.E.M. *P<0.05.

**Fig. 3.**
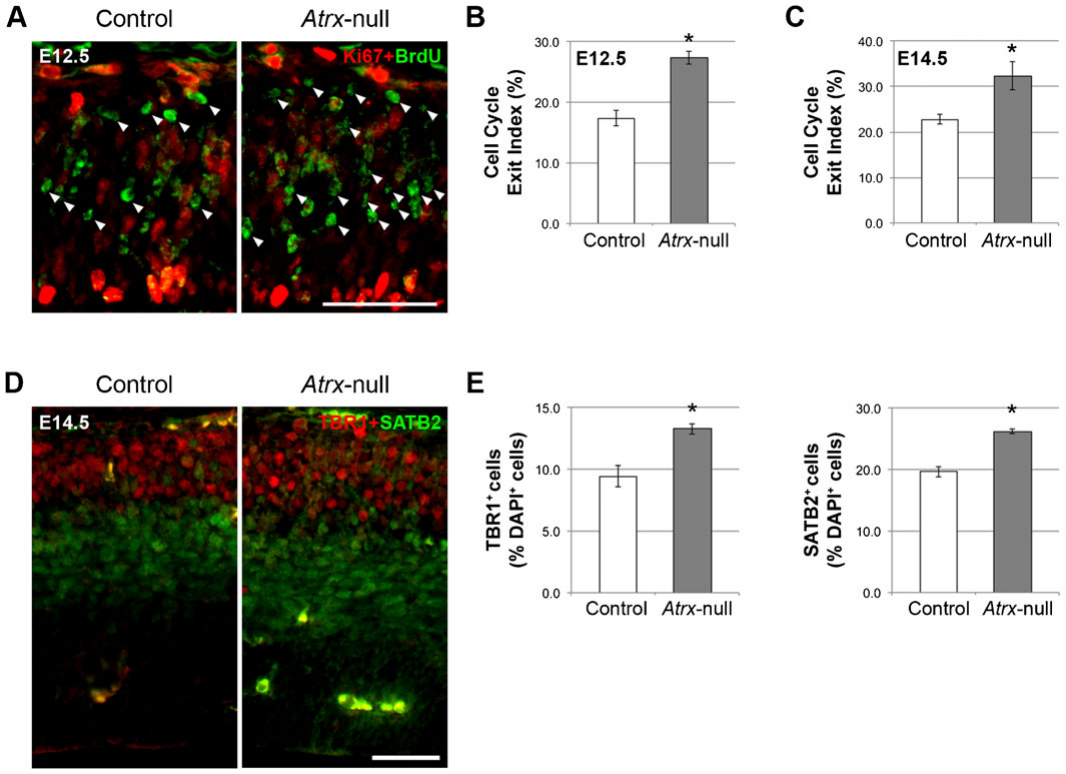
Increased cell cycle exit in the Atrx-null cortex during early neurogenesis results in increased generation of TBR1^+^ and SATB2^+^ early-born neurons. (A) Pregnant female mice were subjected to a 24-hour BrdU pulse prior to sacrifice. Ki67 and BrdU immunostaining in E12.5 (pictured) and E14.5 control and *Atrx*-null cortex. Scale bar: 50 µm. Arrowheads indicate BrdU^+^Ki67^−^ cells that have exited the cell cycle. Cell cycle exit indices were calculated by measuring the ratio of BrdU^+^Ki67^−^ cells to total BrdU^+^ cells in E12.5 (B) and E14.5 (C) control and *Atrx*-null cortex (*n* = 3). (D) TBR1 and SATB2 immunostaining in E14.5 control and *Atrx*-null cortex. Scale bar: 50 µm. (E) Quantification of the proportion of TBR1^+^ and SATB2^+^ cells as a percentage of total DAPI^+^ cells (*n* = 3). Data expressed as mean ± S.E.M. *P<0.05.

Altered postnatal cortical layering could result from precocious differentiation early in neurogenesis, resulting in depletion of the progenitor pool and a failure to generate the correct number of late-born neurons. To determine if the cortical layer abnormalities in the *Atrx*-null cortex were due to increased differentiation of apical progenitor cells, we analyzed the progenitor pool composition, cell cycle exit, and generation of post-mitotic neurons in control and *Atrx*-null cortical cryosections at different developmental time points during the neurogenic period (Figs 2, 3).

We first measured the composition of the progenitor pool by co-immunostaining control and *Atrx*-null cryosections with SOX2 and TBR2, proteins that are specifically expressed in apical/outer radial glia progenitors and basal progenitors, respectively. The relative proportion of SOX2^+^ and TBR2^+^ progenitors was unaffected in the *Atrx*-null cortex at E13.5 (Fig. 2A,B). However we observed a significant decrease in the proportion of SOX2^+^ apical progenitors at E16.5 with a concomitant increase in the proportion of TBR2^+^ basal progenitors located in the subventricular zone and SOX2^+^ outer radial glia progenitors located in the outer subventricular zone (Fig. 2C,D).

Next, we measured the proportion of cells exiting the cell cycle by labeling embryos with BrdU for 24 hours and analyzing Ki67^+^ and BrdU^+^ cells in E12.5 and 14.5 control and *Atrx*-null cortex. The cell cycle exit index (the number of BrdU^+^Ki67^−^ cells divided by the total number of BrdU^+^ cells) was significantly higher in *Atrx*-null cortex compared to controls at E12.5 and E14.5 (Fig. 3A–C), suggesting that *Atrx*-deficiency results in an increased proportion of differentiative divisions during early neurogenesis.

To determine whether increased cell cycle exit resulted in increased differentiation of progenitor cells into post-mitotic neurons, we evaluated the relative proportion of early-born corticothalamic and callosal projection neurons by TBR1 and SATB2 immunostaining in E14.5 control and *Atrx*-null cryosections (Fig. 3D). This analysis demonstrated an increased generation of layer VI TBR1^+^ and early-born SATB2^+^ neurons, which are born at approximately E12.5–E13.5 (Fig. 3E,F). Thus, loss of ATRX causes increased cell cycle exit and enhanced generation of early-born neuronal populations, likely causing depletion of the apical progenitor pool by late neurogenesis.

### Disproportionate cortical layering of the *Atrx*-null cortex

Mutant mice with targeted *Atrx* inactivation in the embryonic mouse forebrain were previously described (*Atrx^Foxg1Cre^* mice, defined as *Atrx*-null hereafter) (Bérubé et al., 2005).

**Fig. 4.**
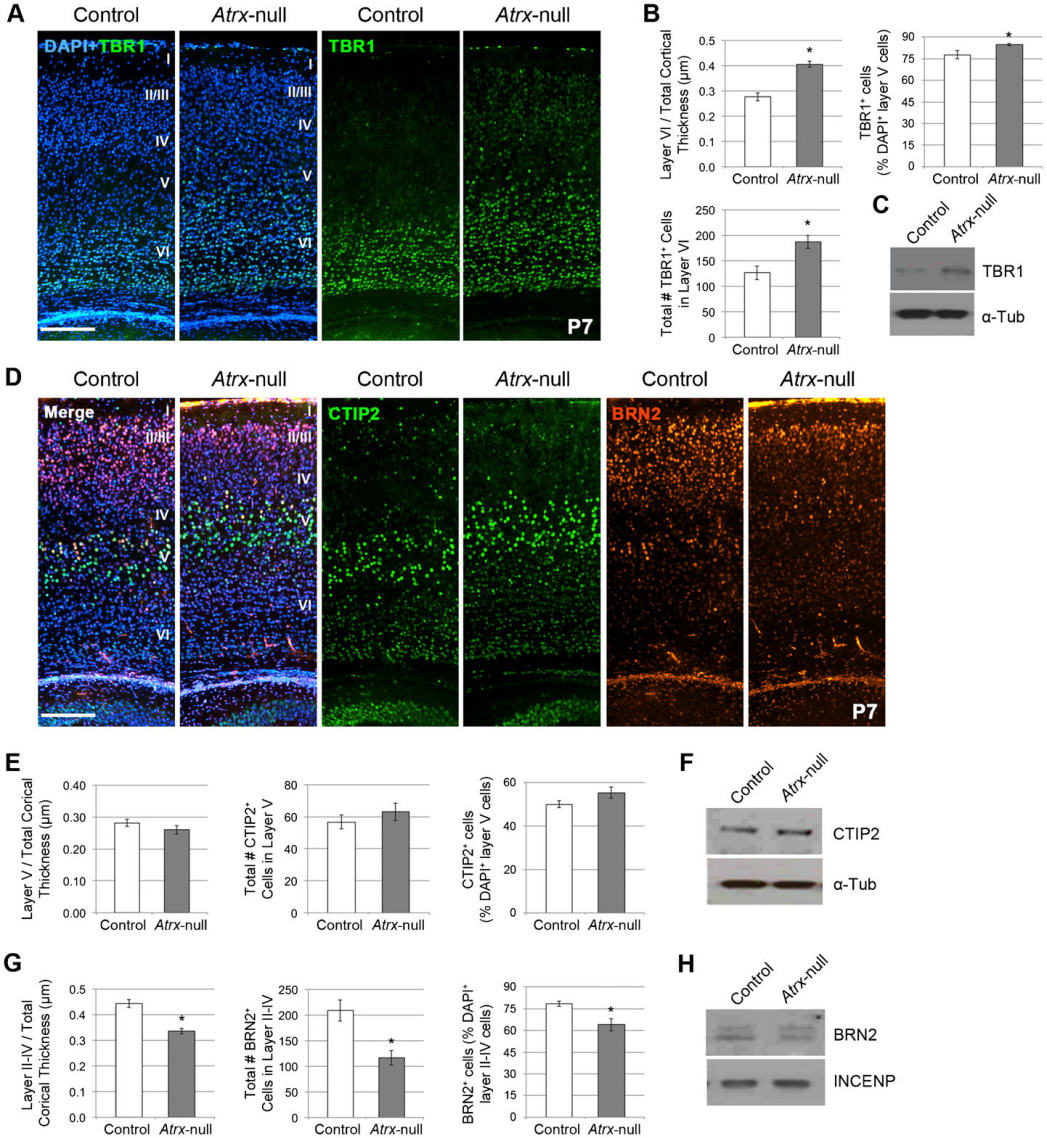
Expansion of cortical layer IV and depletion of cortical layers II–IV in the *Atrx*-null neocortex at P7. (A) TBR1 immunostaining in P7 control and *Atrx*-null cortical sections. Scale bar: 200 µm. (B) Quantification of TBR1^+^ layer VI thickness compared to total cortical thickness, the number, and proportion of TBR1^+^ layer VI neurons (*n* = 5). (C) Western blot analysis of P10 control and *Atrx*-null forebrain tissue. α-Tubulin was used as a loading control. (D) CTIP2 and BRN2 immunostaining in P7 control and *Atrx*-null cortical sections. Scale bar: 200 µm. (E) Quantification of CTIP2^+^ layer V thickness compared to total cortical thickness, the number, and proportion of CTIP2^+^ layer V neurons (*n* = 5). (F) Western blot analysis of P7 control and *Atrx*-null forebrain tissue. α-Tubulin was used as a loading control. (G) Quantification of BRN2^+^ cortical layer II–IV thickness compared to total cortical thickness, the number, and proportion of BRN2^+^ layer II–IV neurons (*n* = 5). (H) Western blot analysis of P10 control and *Atrx*-null forebrain tissue. INCENP was used as a loading control. Data expressed as mean ± S.E.M. *P<0.05.

To investigate the laminar structure of the *Atrx*-null cortex, cryosections from postnatal day (P) 7 control and *Atrx*-null mice were stained with cortical layer markers. The *Atrx*-null cortex had a normal laminar pattern, but significant differences in the thickness and cellularity of several layers (Fig. 4). We used TBR1 as a surrogate marker for layer VI, CTIP2 as a marker of layer V, and BRN2 as a marker of layer II–IV neurons (Bulfone et al., 1995; Leone et al., 2008). Layer VI (TBR1^+^), the deepest and first layer to be generated, was significantly expanded in thickness and cellularity (Fig. 4A,B). Furthermore, TBR1 protein levels appear to be increased in the P7 *Atrx*-null cortex compared to control (Fig. 4C). Layer V (CTIP2^+^) was unchanged in size and cellularity (Fig. 4D,E) and CTIP2 protein levels appear similar in the control and *Atrx*-null cortex (Fig. 4F). Layers II–IV (BRN2^+^), comprised of later-born neurons, were decreased in thickness and cellularity (Fig. 4D,G) however Western blot analysis failed to detect a noticeable difference in BRN2 protein levels between control and *Atrx*-null cortex (Fig. 4H). We previously reported that *Atrx* deletion causes apoptosis in the developing brain, which could explain decreased cellularity in cortical layers II–IV (Bérubé et al., 2005). However, the increase in TBR1-expressing neurons in the postnatal brain cannot be explained by this phenomenon, and indicate an additional mechanism underlies altered neurogenesis in the absence of ATRX.

In summary, the *Atrx*-null cortex exhibits a distinct phenotype, characterized by an expansion of layer VI and a depletion of the upper cortical layers. We put forward evidence for a mechanism involving disruption of the mitotic spindle angle, resulting in increased cell cycle exit and generation of early-born neuron subtypes during early neurogenesis at the expense of late-born neuron subtypes. An additional non-mutually exclusive possibility is that replicative stress and chromosomal abnormalities resulting from *Atrx* deficiency could ultimately influence cell cycle dynamics and result in precocious differentiation, contributing to the phenotypes observed (Bérubé et al., 2005; Ritchie et al., 2008; Watson et al., 2013).

## MATERIALS AND METHODS

### Animal husbandry

Deletion of *Atrx* in the mouse forebrain was achieved by mating *Atrx^loxP^* female mice with heterozygous *Foxg1Cre* knock-in male mice, as previously described (Bérubé et al., 2005). Animal studies were conducted in compliance with the regulations of The Animals for Research Act of the province of Ontario, the guidelines of the Canadian Council on Animal Care, and the policies and procedures approved by the University of Western Ontario Council on Animal Care.

### Cryosectioning

Mouse embryos were collected from CO_2_ euthanized pregnant dams and fixed with 4% paraformaldehyde (PFA) at 4°C overnight. For P7 studies, mice were lethally sedated with CO_2_ and were transcardially perfused with 4% PFA. Samples were cryoprotected in 30% sucrose-PBS, embedded in Shandon Cryomatrix (Thermo Scientific), and sectioned using a Leica cryostat (CM 3050S).

### Immunofluorescence

Cryosections were incubated with primary antibodies diluted in PBS/Triton X-100 overnight at 4°C, rinsed with PBS, and incubated with secondary antibodies for 1 hour. Sections were counterstained with DAPI (1 µg/ml, Sigma) and mounted in SlowFade Gold (Invitrogen) or Vectashield H-1000 (Vector Labs). Antigen retrieval was performed prior to overnight incubation. Antibodies used were anti-TBR1 (1:200, Abcam), anti-CTIP2 (1:500, Abcam), anti-BRN2 (1:50, Santa Cruz), anti-TBR2 (1:200, Abcam), anti-SATB2 (1:200, Abcam), and anti-γ-tubulin (1:200, Sigma). Secondary antibodies used were goat-anti-rabbit Alexa 594 and goat-anti-mouse Alexa 488 (1:800, Invitrogen).

### Microscopy and quantification

Images were captured using a Leica DMI-6000B inverted microscope (Leica Microsystems) equipped with a digital camera (ORCA-ER, Hammamatsu). Image capture was achieved using Openlab v5.0 (Perkin Elmer) and processed using Volocity v5.4 (Perkin Elmer) and Photoshop CS5 (Adobe). To analyze P7 cortical layer thickness, three different measurements separated by 100 µm were obtained perpendicular to the lateral cortical axis in equivalent regions from three sequential cryosections per brain (*n* = 5). For P7 and embryonic cortical cell quantifications, 200 µm-wide columns perpendicular to the lateral cortical axis were quantified for immunopositive cells in equivalent areas from three adjacent cryosections per brain (*n* = 3–5).

### Statistical analysis

Statistical analysis was performed using GraphPad Prism software (GraphPad Software Inc., Version 4.02), and all results are expressed as the mean ± standard error of the mean. *P* values were generated using Student's *t* test (unpaired, two-tailed) to compare between two independent data sets and a value of *P<0.05 was considered statistically significant.

### Western blot analysis

Protein was extracted from forebrain tissue (P7 and P10) using RIPA buffer for 30 min on ice (for TBR1 and CTIP2), or using the NE-PER nuclear protein extraction kit (for BRN2) (Thermo scientific). 10–30 µg of protein was resolved on 8% SDS-PAGE and transferred onto nitrocellulose membranes (Bio-Rad Laboratories). Antibodies used were goat anti-BRN2 (1:1000, Santa Cruz Biotechnology, Inc.), rabbit anti-TBR1 (1:1000, Abcam), and rabbit anti-CTIP2 (1:1000, Abcam) followed by the appropriate horseradish peroxidase (HRP)-conjugated secondary antibody (1:5,000, GE Healthcare). The membranes were incubated in enhanced chemiluminescence reagent (ECL) before exposure to x-ray film (Amersham). The membranes were reprobed with mouse anti-α-tubulin (1:4,000, Sigma–Aldrich) or anti-INCENP as a loading control.

### Cell cycle exit analysis

Pregnant mice were injected intraperitoneally with cell proliferation labeling reagent (10 mM BrdU and 1 mM FdU in H_2_O) at 1 ml per 100 g body weight (GE Healthcare Life Sciences) and embryos were collected and processed for immunofluorescence analysis 24 hours later. Prior to immunofluorescence analysis, cryosections were treated with 2 N HCl to denature the DNA and neutralized with 0.1 M Na_2_B_4_O_7_, pH 8.5.

### Neuroprogenitor cultures

Embryonic cortices (E13.5) were dissected, dissociated by trituration and plated in Neurobasal medium (Gibco) supplemented with 1% Penicillin-Streptomycin, 1% Glutamax (Gibco), 1% N2 supplement, and 25 ng/ml bFGF. Cultures were maintained in a humidified tissue culture incubator at 37°C and 5% CO_2_.
